# Evaluation of a combined respiratory‐gating system comprising the TrueBeam linear accelerator and a new real‐time tumor‐tracking radiotherapy system: a preliminary study

**DOI:** 10.1120/jacmp.v17i4.6114

**Published:** 2016-07-08

**Authors:** Takehiro Shiinoki, Shinji Kawamura, Takuya Uehara, Yuki Yuasa, Koya Fujimoto, Masahiro Koike, Tatsuhiro Sera, Yuki Emoto, Hideki Hanazawa, Keiko Shibuya

**Affiliations:** ^1^ Department of Radiation Oncology Graduate School of Medicine, Yamaguchi University Yamaguchi Japan; ^2^ Department of Radiological Technology Yamaguchi University Hospital Yamaguchi Japan

**Keywords:** respiratory motion, real‐time tumor‐tracking, respiratory‐gated radiotherapy

## Abstract

A combined system comprising the TrueBeam linear accelerator and a new real‐time, tumor‐tracking radiotherapy system, SyncTraX, was installed in our institution. The goals of this study were to assess the capability of SyncTraX in measuring the position of a fiducial marker using color fluoroscopic images, and to evaluate the dosimetric and geometric accuracy of respiratory‐gated radiotherapy using this combined system for the simple geometry. For the fundamental evaluation of respiratory‐gated radiotherapy using SyncTraX, the following were performed: 1) determination of dosimetric and positional characteristics of sinusoidal patterns using a motor‐driven base for several gating windows; 2) measurement of time delay using an oscilloscope; 3) positional verification of sinusoidal patterns and the pattern in the case of a lung cancer patient; 4) measurement of the half‐value layer (HVL in mm AL), effective kVp, and air kerma, using a solid‐state detector for each fluoroscopic condition, to determine the patient dose. The dose profile in a moving phantom with gated radiotherapy having a gating window ≤4 mm was in good agreement with that under static conditions for each photon beam. The total time delay between TrueBeam and SyncTraX was <227 ms for each photon beam. The mean of the positional tracking error was <0.4 mm for sinusoidal patterns and for the pattern in the case of a lung cancer patient. The air‐kerma rates from one fluoroscopy direction were 1.93±0.01, 2.86±0.01, 3.92±0.04, 5.28±0.03, and 6.60±0.05 mGy/min for 70, 80, 90, 100, and 110 kV X‐ray beams at 80 mA, respectively. The combined system comprising TrueBeam and SyncTraX could track the motion of the fiducial marker and control radiation delivery with reasonable accuracy; therefore, this system provides significant dosimetric improvement. However, patient exposure dose from fluoroscopy was not clinically negligible.

PACS number(s): 87.53.Bn, 87.55.km, 87.55.Qr

## I. INTRODUCTION

In radiation therapy, tumor motion during respiration results in significant geometric and dosimetric uncertainties in the dose delivery to the target in the thorax and abdomen. The peak‐to‐peak magnitude of the respiratory motion is as large as 20−30 mm.^(1)^ Conventionally, large internal margins (IMs) are needed for fully covering the geometric changes that occur during free breathing; these large IMs may result in toxicity to healthy tissue.

As techniques for managing respiratory‐induced tumor movement, breath‐holding,^(2)^ respiratory‐gated radiotherapy,^3^, ^4^, ^5^ and dynamic tumor tracking delivery techniques^(6)^ are effective in reducing the IMs, resulting in a lower dose to the normal tissue and, consequently, a lower risk of complications. To use these techniques in clinical practice, a correlation between external markers or sensors and internal tumor motion is needed. Many researchers^7^, ^8^, ^9^ have shown a correlation between the external marker and the internal tumor motion, and have reported that the maximum variations between the internal motion and external markers were about 10 mm. Although a correlation between external markers and internal tumor positions exists, external markers cannot be used as an adequate indicator to determine the internal tumor position in some patients.

Ge et al.^(10)^ have reported the observation of intrafraction motion variation of the abdominal tumor in a CT simulation and during the course of radiation treatment. They suggested that it is important to monitor the intrafraction motion during treatment delivery.

**Figure 1:**
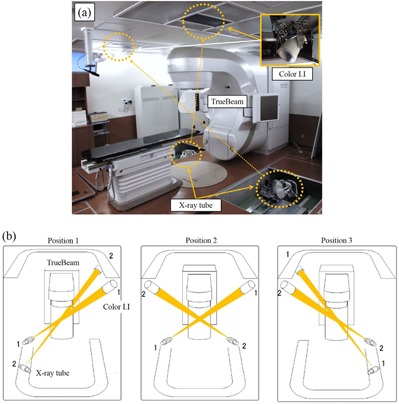
Photograph of combined system (a) comprising TrueBeam and SyncTraX at the Yamaguchi University hospital. SyncTraX consists of two X‐ray tubes and two color image intensifiers (I.I.s). Using the X‐ray tubes and color I.I.s along two directions (b), the position of a fiducial marker close to the tumor is automatically extracted using a pattern recognition technique to calculate the three‐dimensional (3D) coordinates for color fluoroscopic images.

Therefore, our group has aimed to achieve respiratory‐gated radiotherapy of respiratory‐induced mobile tumors with a novel system that combined TrueBeam (Varian Medical Systems, Palo Alto, CA) and a new real‐time, tumor‐tracking radiotherapy (RTRT) system, SyncTraX (Shimadzu Co., Kyoto, Japan) (Fig.1(a)). TrueBeam is a new linear accelerator (linac) model that has the capacity to deliver both traditional flattened (FF) photon beams and flattening filter‐free (FFF) photon beams. The concepts of SyncTraX are almost the same as those of the previous Mitsubishi‐developed RTRT system (Mitsubishi Electronics Co., Ltd., Tokyo, Japan).^(11)^ This system consists of two color image intensifiers (I.I.s) and X‐ray tubes. The position of X‐ray tubes and color I.I.s can be selected from three options; these positions are indicated in Fig. 1(b). Therefore, the fiducial markers could be observed using fluoroscopy in radiation treatment with noncoplanar beams. The color fluoroscopic images have a wide dynamic range that reduces the halation of the lung and improves the brightness of fluoroscopic images of the heart and spine regions.^(12)^

**Figure 2:**
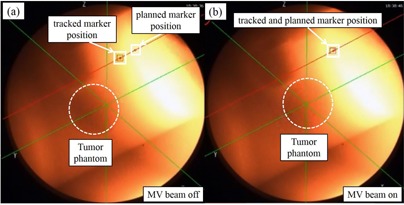
Example of color fluoroscopic image from one X‐ray tube: (a) when the actual fiducial marker's position is not within several millimeters of the planned 3D marker's position, the treatment MV beam is turned off; (b) when the actual fiducial marker's position is within several millimeters of the planned 3D marker's position, the MV treatment beam is turned on and the delivery proceeds.

Using color fluoroscopic images acquired along two directions, the position of a fiducial marker close to a tumor is automatically extracted using a pattern recognition technique to calculate the three‐dimensional (3D) coordinates of the color fluoroscopic images. When the tracked fiducial marker comes within several millimeters (gating window) of the 3D planned position of the fiducial marker, the megavoltage (MV) treatment beam is turned on (Fig. 2). This system uses a spatial gating technique that gates the beam by way of the absolute 3D position of the internal fiducial marker, instead of using an external surrogate such as that used in the phase or amplitude. It is necessary to verify the accuracy of this combined system before clinical implementation. In this study, we evaluated the dosimetric and geometric accuracy of respiratory‐gated radiotherapy using this combined system.

## II. MATERIALS AND METHODS

### A. Verification of respiratory‐gated radiotherapy using SyncTraX

For the fundamental evaluation of respiratory gated radiotherapy using SyncTraX, the following were performed: 1) determination of dosimetric and positional characteristics of sinusoidal patterns using a motor‐driven base for several gating windows; 2) measurement of time delay using an oscilloscope; 3) positional verification of sinusoidal patterns and the pattern in the case of a lung cancer patient; 4) measurement of the half‐value layer (HVL in mm AL), effective kVp, and air kerma, using a solid‐state detector for each fluoroscopic condition, to determine the patient dose. As the differences in the position of fiducial markers that can be measured using each position of fluoroscopy have been corrected to <1 mm, all the above verifications were performed using fluoroscopy position 2 (Fig. 1(b)).

### B. Dosimetric and positional characteristics of sinusoidal patterns using a motor‐driven base for several gating windows

Measurements were performed on a well‐commissioned TrueBeam linear accelerator, which has a 120‐leaf independently moving MLC with a 5‐mm leaf width at the isocenter for the 20 cm central field.

**Figure 3:**
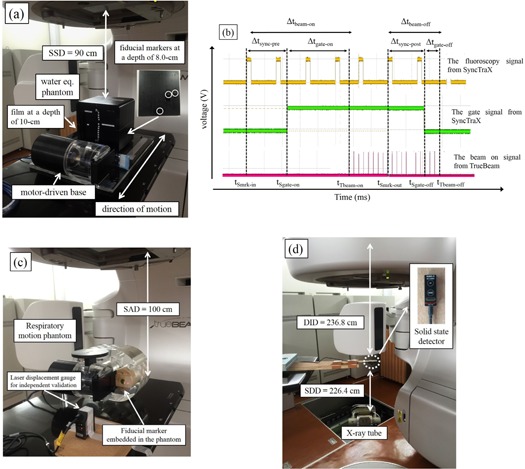
Photograph of the experimental setup (a) for determining dosimetric and positional characteristics of sinusoidal patterns using a motor‐driven base for several gating windows. Example of an oscilloscope image (b) for respiratory‐gated radiotherapy using SyncTraX. The yellow, green, and pink lines show the fluoroscopic signal from SyncTraX, the gate signal from SyncTraX, and the beam‐on signal from TrueBeam, respectively. Photograph of the experimental setup (c) for the positional verification of sinusoidal patterns and the pattern in the case of a lung cancer patient. Photograph of the experimental setup (d) for the measurement of air kerma, kVp, and half‐value layer (HVL in mm AL) using a solid‐state detector for various fluoroscopic conditions.

The experimental setup is shown in Fig. 3(a). The water‐equivalent phantom (RT‐3000‐New; R‐Tech, Tokyo, Japan) was made of acrylonitrile‐butadiene‐styrene resin (ρ approx. equal to $1.05{\text{cm}}^{3}$) with dimensions of $20\times 20\times 17\;{\text{cm}}^{3}$ and was set on a motor‐driven base (QUASAR Programmable Respiratory Motion Platform; Modus Medical, London, ON, Canada). The source‐to‐surface distance was 90 cm. EBT3 Gafchromic film (International Specialty Products, Wayne, NJ) was placed perpendicular to the beam axis at a depth of 10 cm. Four markers were made on the film to identify the isocenter. Additionally, three fiducial markers with a diameter of 1.5 mm (FMR‐201CR; Olympus Co., Ltd., Tokyo, Japan) were placed at a depth of 8 cm. The EBT3 Gafchromic film was irradiated under static, gating, and nongating states for the sinusoidal pattern (Amplitude [A]:20 mm, breathing period [T]:4 s) in the craniocaudal (CC) direction. A single photon beam was set at a gantry angle of 0°. The field size was $50\times 50\;{\text{mm}}^{2}$. The photon beam energies were set to 6 MV‐FF, 10 MV‐FF, 6 MV‐FFF, and 10 MV‐FFF. The monitor unit (MU) was set to 200 MU. The dose rates were set to 300 MU/min (6 MV‐FF, 10 MV‐FF), 600 MU/min (6 MV‐FFF), and 1200 MU/min (10 MV‐FFF), which are the maximum dose rates that can be used in this combined system. The tube current and voltage of fluoroscopy were set to 63 mA and 80 kV, respectively. These conditions enable us to recognize the fiducial marker in the water‐equivalent phantom.

For respiratory‐gated irradiation, the gating window was set to 2, 4, 6, 8, and 10 mm. In total, 20 irradiated films (two energies (6 MV,10 MV)×two types of photon beams (FF or FFF)×five gating windows (sizes mentioned above) were scanned in the same orientation (ES‐10000G; Epson Corp., Nagano, Japan) with a resolution of 72 dpi in 48‐bit color scale with a 24 h postexposure period. All the films were analyzed using commercially available radiation dosimetry software (DD system, ver.10.12; R'Tech Inc., Tokyo, Japan). The dose distribution was normalized to the maximum dose by using a calibration curve to reduce film uncertainties, and then, the static, nongating, and several gating data were compared for an area receiving more than 50% isodose to evaluate the dose using the gamma index with a dose difference/distance‐to‐agreement criterion ($\gamma_{D\%/\text{dmm}}$) of 2%/2 mm. The relationship between gamma pass rate of $\gamma_{2\%/2\;\text{mm}}$ and gating windows was analyzed.

### C. Evaluation of time delay using oscilloscope

Time delay measurement was performed using an oscilloscope (DSO6054A, Agilent Technologies Inc., Santa Clara, CA), which was used to measure the voltage from the TrueBeam and SyncTraX signal during the respiratory gated radiotherapy at 6 MV‐FF, 6 MV‐FFF, 10 MV‐FF, and 10 MV‐FFF.

Figure 3(b) shows a schematic of the oscilloscope image during respiratory‐gated radiotherapy using SyncTraX. The yellow line corresponds to the fluoroscopic signal from SyncTraX, the green line corresponds to the gate signal from SyncTraX, and the pink line corresponds to the beam‐on signal from TrueBeam.

The beam‐on delay ($\Delta t_{\text{beam}‐\text{on}}$) was defined as $\Delta t_{\text{syn}‐\text{pre}}+\Delta t_{\text{gate}‐\text{on}}$.

(1) $$\Delta t_{syn-pre}=t_{Sgate-on}-t_{Smrk-in}$$

and

(2) $$\Delta t_{gate-on}=t_{Tbeam-on}-t_{Sgate-on}$$

where $t_{Smrk-in}$ is the time at the signal from SyncTraX when the tracked fiducial marker arrives within the 2 mm gating window, $t_{Sgate-on}$ is the time at the gate‐on signal from SyncTraX, and $t_{Tbeam-on}$ is the time at the beam‐on signal from TrueBeam.

The beam‐off delay ($\Delta t_{\text{beam}‐\text{off}}$) was defined as $\Delta t_{\text{syn}‐\text{post}}+\Delta t_{\text{gate}‐\text{off}}$.

(3) $$\Delta t_{syn-post}=t_{Sgate-off}-t_{Smrk-out}$$

and

(4) $$\Delta t_{gate-off}=t_{Tbeam-off}-t_{Sgate-off}$$

where $t_{Smrk-out}$ is the time at the signal from SyncTraX when the tracked fiducial marker goes outside the 2 mm gating window, $t_{Sgate-off}$ is the time at the gate‐off signal from SyncTraX, and $t_{Tbeam-off}$ is the time at the beam‐off signal from TrueBeam.

### D. Positional verification of sinusoidal patterns and the pattern in the case of a lung cancer patient

The experimental setup is shown in Fig. 3(c). The fiducial marker embedded in the Quasar respiratory motion phantom (Modus Medical) was moved using sinusoidal patterns ([A]:20 mm, [T]:2,4 s) and, in the case of a lung cancer patient, in the CC direction. The tracking accuracy was verified using a laser displacement gauge (IL‐300; Keyence Corp., Osaka, Japan) with a positional accuracy of 0.05 mm. The laser displacement gauge was used an independent validation of tracking accuracy of SyncTraX and was not part of SyncTraX. In the experiment, the position of the phantom embedded the fiducial marker was measured with the laser displacement gauge every 10 ms for independent validation. Simultaneously, the fiducial marker position was measured using SyncTraX every 30 ms. The time interval between the laser displacement gauge measurements and those acquired using SyncTraX were matched to calculate the positional tracking error.

The positional tracking error was defined as

(5) $$E_{p}=-y_{ml}-y_{ms}$$

where $y_{\text{ml}}$ is the phantom position in the CC direction, measured with the laser displacement gauge, and $y_{\text{ms}}$ is the fiducial marker position in the CC direction, measured with SyncTraX. The mean ± SD values of the absolute $E_{p}$ value were calculated for the sinusoidal patterns and the pattern in the case of a lung cancer patient.

### E. Half‐value layer (HVL in mm AL), effective kVp, and air kerma measured using solid‐state detector for various fluoroscopic conditions

The experimental setup is shown in Fig. 3(d) and comprises a rotating anode X‐ray tube assembly (0.6/1 J317c‐282(Y), Shimadzu, Kyoto, Japan).

The solid‐state detector (AGMS‐D, Radcal Accu‐Gold, Monrovia, CA) was annually calibrated by the Japan Quality Assurance Organization, and was set at the isocenter. This detector is generally designed for diagnostic radiography.^(13)^ The source‐to‐detector distance (SDD) was 226.4 cm, and the detector‐to‐imager distance (DID) was 236.8 cm. The solid‐state detector was set perpendicular to the beam axis from the X‐ray tube. The diameter of the field of view for fluoroscopy measurements was about 11 cm at the isocenter. The HVL, effective kVp, and air kerma were measured with the solid‐state detector for 60 s. The tube voltage was set to 70, 80, 90, 100, or 110 kVp, and the tube current was set to 32, 40, 50, 63, 80, 90, or 100 mA, respectively. The frame rate was set to 30 frames per second, and the pulse duration was 2.8 ms. All the measurements were performed along one fluoroscopy direction. The HVL, effective kVp, and air kerma were calculated as the mean ± SD values from three measurements for each current and voltage of the X‐ray tube.

## III. RESULTS

### A. Dosimetric and positional characteristics of sinusoidal patterns using a motor‐driven base for several gating windows

**Figure 4:**
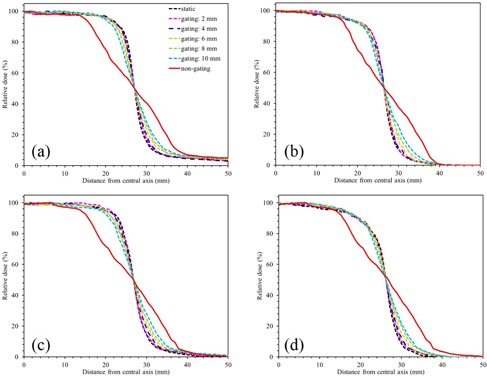
Dose profiles of a $50\times 50\;{\text{mm}}^{2}$ field under static condition, gating with several gating windows, and nongating states for a sinusoidal pattern: (a) 6 MV‐FF, (b) 6 MV‐FFF, (c) 10 MV‐FF, and (d) 10 MV‐FFF. The gating delivery had gating windows of 2, 4, 6, 8, and 10 mm. The label “non‐gating” indicates nongating delivery measured on a respiratory motion phantom, while “static” corresponds to a static phantom for the nongating beam. To compare the profiles, all dose profiles are shifted to align their radiation field edge (50% level) with the static profile.

Figure 4 shows the dose profiles of the $50\times 50\;{\text{mm}}^{2}$ field under static, gating, and nongating states for a sinusoidal pattern. The “non‐gating” data were for the nongating delivery measured on the respiratory motion phantom, while the “static” data were acquired on the static phantom for the nongating beam. To compare the profiles, all the dose profiles were shifted to align their radiation field edge (50% level) with the static profile.^(14)^ For the case of a 2 mm gating window, the blurring effect due to the phantom motion was substantially reduced. On the other hand, for the case of a 10 mm gating window, geometric changes were caused by residual motion.

**Figure 5:**
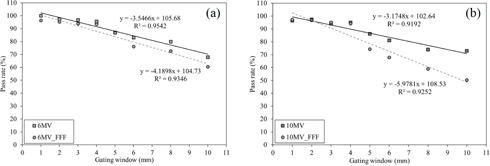
Relationship between the gamma pass rate of $\gamma_{2\%/2\;\text{mm}}$ and gating windows for (a) 6 MV‐FF and 6 MV‐FFF photon beams and (b) 10 MV‐FF and 10 MV‐FFF photon beams. When the gating window was ≤4 mm, the gamma pass rate of of $\gamma_{2\%/2\;\text{mm}}$ was ≥90% for each photon beam. On the other hand, when the gating window was >4 mm, the gamma pass rate of $\gamma_{2\%/2\;\text{mm}}$ was ≤90% for each photon beam.

Figure 5 shows the relationship between the gamma pass rate of $\gamma_{2\%/2\;\text{mm}}$ and gating windows. When the gating window was ≤4 mm, the gamma pass rate of $\gamma_{2\%/2\;\text{mm}}$ was ≥90% for each photon beam. On the other hand, when the gating window was >4 mm, the gamma pass rate of $\gamma_{2\%/2\;\text{mm}}$ was ≤90% for each photon beam. Especially, the residual motion in the gating window affects the gamma pass rate for the 10 MV‐FFF photon beam.

### B. Evaluation of time delay using oscilloscope

The mean ± SD values of $\Delta t_{\text{sync}‐\text{pre}}$ were 45.3±0.5 ms, 45.6±0.5 ms, 45.2±0.4 ms, and 45.7±0.4 ms in the case of 6 MV‐FF, 6 MV‐FFF, 10 MV‐FF, and 10 MV‐FFF photon beams, respectively. The mean ± SD values of $\Delta t_{\text{gate}‐\text{on}}$ were 109.5±11.8 ms, 103.5±8.6 ms, 115.9±12.1 ms, and 112.1±15.3 ms in the case of 6 MV‐FF, 6 MV‐FFF, 10 MV‐FF, and 10 MV‐FFF photon beams, respectively. The mean ± SD values of $\Delta t_{\text{sync}‐\text{post}}$ were 44.2±0.3 ms, 44.2±0.7 ms, 44.4±0.7 ms, and 42.0±0.0 ms in the case of 6 MV‐FF, 6 MV‐FFF, 10 MV‐FF, and 10 MV‐FFF photon beams, respectively. The mean ± SD values of $\Delta t_{\text{gate}‐\text{off}}$ were 27.3±12.0 ms, 18.5±14.4 ms, 23.7±7.2 ms, and 27.0±10.7 ms in the case of 6 MV‐FF, 6 MV‐FFF, 10 MV‐FF, and 10 MV‐FFF photon beams, respectively. For each photon beam, $\Delta t_{\text{beam}‐\text{on}}+\Delta t_{\text{beam}‐\text{off}}<227\;\text{ms}$.

### C. Positional verification of sinusoidal patterns and the pattern in the case of a lung cancer patient

**Figure 6:**
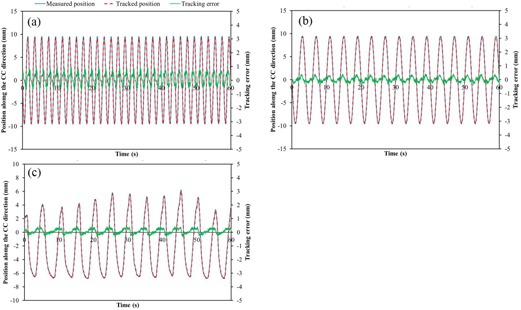
Variations in the measured and tracked positions of the fiducial marker for sinusoidal patterns with (a) A=20 mm, T=2 s, and (b) A=20 mm, T=4 s; (c) the pattern in the case of a lung cancer patient. For the sinusoidal patterns, the mean ± SD values of absolute $E_{p}$ were 0.31±0.20 mm (A=20 mm, T=2 s) and 0.13±0.09 mm (A=20 mm, T=4 s), and that for pattern in the case of the lung cancer patient was 0.16±0.10 mm. The blue line and red dashed line show the measured and tracked positions of the fiducial marker, respectively, while the green line shows the positional tracking error.

Figure 6 shows the variations in the measured and tracked positions of the fiducial marker for the sinusoidal pattern with a peak‐to‐peak amplitude of 20 mm and breathing period of 2 or 4 s. For sinusoidal patterns, the mean ± SD values of absolute $E_{p}$ were 0.31±0.20 mm (A=20 mm, T=2 s) and 0.13±0.09 mm (A=20 mm, T=4 s), and that for the lung cancer patient was 0.16±0.10 mm.

### D. Half‐value layer (HVL in mm AL), effective kVp, and air kerma measured using solid‐state detector for various fluoroscopic conditions

For nominal 70, 80, 90, 100, and 110 kV X‐ray beams at 63 mA, the HLVs for each kilovoltage of the X‐ray tube were 3.22±0.02, 3.70±0.01, 4.15±0.00, 4.60±0.01, and 5.02±0.01 mm, respectively, and the effective kVp values were 68.03±0.06, 78.30±0.30, 89.60±0.10, 100.90±0.26, and 111.90±0.36 kVp, respectively.

**Figure 7:**
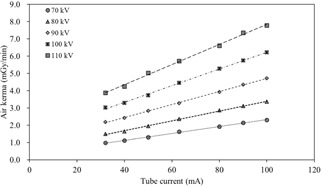
Relationship between the tube current of the X‐ray tube and air‐kerma rate along one fluoroscopy direction. The air‐kerma rates were 1.93±0.01, 2.86±0.01, 3.92±0.04, 5.28±0.03, and 6.60±0.05 mGy/min for the nominal 70, 80, 90, 100, and 110 kV X‐ray beams at 80 mA, respectively.

Figure 7 shows the relationship between the current of the X‐ray tube and air‐kerma rate (mGy/min) from the X‐ray tube for each kilovoltage of the X‐ray tube. The air kerma rate increased steeply with the increase in the tube current. The air‐kerma rates along one fluoroscopy direction were 1.93±0.01, 2.86±0.01, 3.92±0.04, 5.28±0.03, and 6.60±0.05 mGy/min for the nominal 70, 80, 90, 100, and 110 kV X‐ray beams at 80 mA, respectively.

## IV. DISCUSSION

This study evaluated the dosimetric accuracy, geometric accuracy, time delay, and patient dose associated with the use of the respiratory‐gating system that combined the TrueBeam and SyncTraX system for the first time. We found that these systems could reliably deliver gated treatment.

In the current study, all dose profiles were obtained at a gantry angle of 0° because it was necessary to evaluate the fundamental performance of this combined system, which was installed for the first time in Japan.

For a smaller gating window, the blurring effect due to phantom motion was substantially reduced. On the other hand, for a larger gating window, geometric changes were caused by residual motion. The results of our study indicate that a residual motion of 4 mm should be used for respiratory‐gated radiotherapy using the combined system of TrueBeam and SyncTraX for each photon beam (Fig. 5). High dosimetric and geometric accuracy were needed to implement stereotactic body radiotherapy (SBRT) using respiratory gating in clinical practice;^(15)^ therefore, a dose difference/distance‐to‐agreement criterion of 2%/2 mm was used in this present study. In this study, we verified the optimal gating window by using a phantom moved in a regular pattern. However, patient breathing is not similar to a moving phantom. Other factors exist in the case of a real patient, such as the change in the breathing pattern or baseline shift, which may introduce additional errors to the delivery.

In this study, the time delay was measured using an oscilloscope, and the total time delay was <227 ms for each photon beam. Some researchers reported time delays measured using films.^16^, ^17^ The measurement methods involving an oscilloscope help avoid human errors in positioning the film in the measurement setup. McCabe and Wiersma^(18)^ suggested that the measurement methods using an oscilloscope can be used to measure the time delay for respiratory gating with millisecond‐order temporal resolutions. We applied this method to measure the delay time of combined respiratory‐gating system of TrueBeam and SyncTraX. Table 1 compares time delays of various respiratory‐gating systems. Chang et al.^(19)^ reported that the time delay of linac‐based ExacTrac (Brainlab, Feldkirchen, Germany) and the real‐time position management (RPM) (Varian Medical Systems, Palo Alto, CA) gating system were 200±30 ms and 90±10 ms, respectively. Smith et al.^(20)^ reported that the time delay of the combined gating system of Trilogy (Varian Medical Systems) and Calypso (Calypso Medical, Seattle, WA) was 75.0±12.7 ms for beam on and 65.1±12.9 ms for beam off. Woods and Rong^(21)^ measured the time delay of the TrueBeam and RPM gating system using an oscilloscope with millisecond‐order temporal resolution and reported that the time delay for MV beam on was 139±10 ms.

**Table 1:** Comparison of delay times of various respiratory‐gating systems

*Author*	*Modality*	$\Delta t_{beam‐on}$ *(ms)*	$\Delta t_{beam‐off}$ *(ms)*
Chang et al.^(19)^	Novalis Tx+ExacTrac	200±30	
	Novalis Tx+RPM	90±10	
Smith et al.^(20)^	Trilogy+Calypso	75.0±12.7	65.1±12.9
Woods and Rong^(21)^	TrueBeam+RPM	139±10	
AAPM TG142^(22)^		100	100
This study	TrueBeam+SyncTraX		
	6 MV‐FF	154.8±12.1	71.5±12.1
	6 MV‐FFF	161.4±12.1	62.7±14.1
	10 MV‐FF	148.7±8.6	68.1±7.2
	10 MV‐FFF	157.8±15.3	69.0±10.7

RPM=real‐time positioning management; FF=flattened; FFF=flattening filter‐free.

Although the measurement methodology and system used in the previous studies are different from those in the present study, the results acquired in this study are comparable to those reported previously. However, according to a report published by the American Association of Physicists in Medicine (AAPM),^(22)^ it was recommended that $\Delta t_{\text{beam}‐\text{on}}$ and $\Delta t_{\text{beam}‐\text{off}}$ be ≤100 ms. Our $\Delta t_{\text{beam}‐\text{on}}$ results were slightly longer than the AAPM recommended time delay. Sharp et al.^(23)^ reported that a prediction algorithm of respiratory tumor motion was needed to perform respiratory‐gated radiotherapy with systems that have time delays of 200 ms or greater. However, respiratory‐gated radiotherapy might be performed with high accuracy using this combined system without a prediction algorithm of respiratory tumor motion.

Our results show that marker positions acquired using SyncTraX are in good agreement with those acquired using a laser displacement gauge. Ono et al.^(24)^ evaluated the tracking accuracy of the gimbal X‐ray head of Vero4 DRT (Mitsubishi Heavy Industries and Brainlab) were evaluated by using a laser displacement gauge to avoid the driving error of a respiratory motion phantom. We applied this method to measure the tracking accuracy of SyncTraX. The means of the positional tracking error were <0.4 mm for the sinusoidal pattern and for the pattern in the case of a lung cancer patient. When the marker speed is greater, the positional tracking error tends to increase slightly (Figs. 6(a) and (b)). Even for the pattern acquired under severe motion, SyncTraX tracked the fiducial marker in real time with high accuracy. Our results indicate that the SyncTraX has high tracking accuracy, even for a moving target. Table 2 compares the tracking accuracies of various respiratory gating system. Chang et al.^(19)^ reported that the average mean discrepancies in the positional tracking errors between the ExacTrac system and references were 1.0±1.6 mm and 1.9±3.2 mm for sine and patient respiratory patterns, respectively. For the RPM system, these were 0.9±1.6 mm and 1.7±2.6 mm for sine and patient respiratory patterns, respectively. Our results acquired using SyncTraX are comparable to their results, which demonstrates the high tracking accuracy for respiratory gating using the internal fiducial marker.

Table 3 compares the air kerma of the previous RTRT system with that of the SyncTraX system. The air kerma measured in this study were recalculated at 2.0 ms pulse duration for comparison with the previous report. Our results acquired using SyncTraX were slightly lower than the results acquired using the previous RTRT system. Shirato et al.^(25)^ reported that the air kerma at 80 mA from fluoroscopy was 238.80±0.54 mGy/h for a pulse width of 2.0 ms with a nominal 100 kV X‐ray beam, and intensity‐modulated radiotherapy (IMRT) using the previous RTRT system may result in an unacceptably high radiation dose to the skin surface and possibly to deep tissues. The previous RTRT system is capable of only delivering dose with a 6 MV‐FF photon beam at 150 MU/min and with a 10 MV‐FF photon beam at 200 MU/min.^(6)^ Thus, the time of delivering the planned dose is long, which increases the exposure dose, especially in case of IMRT and SBRT. In the present study, we evaluated the respiratory gating with TrueBeam and SyncTraX. TrueBeam has the capability of radiation delivery via FFF beams. Prendergast et al.^(26)^ reported that the treatment time with FFF linacs is lower by more than 50% compared to that with conventional linacs. Therefore, the patient exposure dose might be reduced by reducing the fluoroscopic irradiation time. Monochromatic I.I.s were used for the previous RTRT system. A high voltage and current for fluoroscopy were needed to identify the fiducial marker with high accuracy. On the other hand, SyncTraX used the color I.I. to determine the position of the fiducial marker. The color I.I. has a much wider dynamic range and higher sensitivity than the monochromatic I.I.; therefore, it could be possible to maintain a high identification rate of the fiducial marker at a lower X‐ray exposure.^(27)^ Consequently, the exposure dose could be reduced for patients. In future work, it is proposed to determine the optimal fluoroscopy condition to reduce the exposure dose.

**Table 2:** Comparison of tracking accuracies of various respiratory‐gating systems

*Author*	*Modality*	*Sinusoidal Pattern Difference (mm)*	*Patient Pattern Difference (mm)*
Chang et al.^(19)^	Novalis Tx+ExacTrac Novalis Tx+RPM	1.0±1.6 ([A]:28 mm, [T]:5 s) 0.9±1.6 ([A]:28 mm, [T]:5 s)	1.9±3.2 1.0±1.6
This study	TrueBeam+SyncTraX	0.3±0.2 ([A]:20 mm, [T]:2 s) 0.1±0.1 ([A]:20 mm, [T]:4 s)	0.2±0.1

RPM=real‐time positioning management; A=amplitude; T=breathing period.

**Table 3:** Comparison of the air kerma of the previous RTRT system with that of the SyncTraX system

			*Air Kerma@Isocenter (mGy)*	
*kV*	*mA*	*ms*	*Shirato et al*.^(25)^	*This Study*
80	80	2	2.45	2.04
100	80	2	4.35	3.77

## V. CONCLUSIONS

We evaluated respiratory gating using the TrueBeam linear accelerator and the SyncTraX system. Our results indicated that this combined system could track the motion of a fiducial marker and control radiation delivery with reasonable accuracy; therefore, this system provided significant dosimetric improvement. However, the patient exposure dose from fluoroscopy was not clinically negligible.

## ACKNOWLEDGMENTS

This research was supported by the Japan Society for the Promotion of Science (JSPS) KAKENHI Grant Number 15K21194 (T.S.). The authors wish to thank Dr. Yusuke Aita and Seiji Yamanaka of Shimadzu Corporation for help with the time‐delay measurements.

## COPYRIGHT

This work is licensed under a Creative Commons Attribution 3.0 Unported License.

## Supporting information

Supplementary Material
