# “Evaluation of a combined respiratory‐gating system comprising the TrueBeam linear accelerator and a new real‐time tumor‐tracking radiotherapy system: A preliminary study” [JACMP, 17(4), 2016]

**DOI:** 10.1002/acm2.12125

**Published:** 2017-06-26

**Authors:** T Shiinoki, S Kawamura, T Uehara, Y Yuasa, K Fujimoto, M Koike, T Sera, Y Emoto, H Hanazawa, K Shibuya

After the publication of the manuscript titled “Evaluation of a combined respiratory‐gating system comprising the TrueBeam linear accelerator and a new real‐time tumor‐tracking radiotherapy system: a preliminary study”^1^, an error was discovered in measured delay time (Δt_gate‐on_) and Table 1 (Δt_beam‐on_).

**Table 1:** Comparison of delay times of various respiratory gating systems

Author	modality	▵t_beam‐on_ (ms)	▵t_beam‐off_ (ms)
Chang et al.^(19)^	Novalis Tx + ExacTrac	200 ± 30	
	Novalis Tx + RPM	90 ± 10	
Smith et al.^(20)^	Trilogy + Calypso	75.0 ± 12.7	65.1 ± 12.9
Woods et al.^(21)^	TrueBeam + RPM	139 ± 10	
AAPM TG142^(22)^		100	100
This study	TrueBeam + SyncTraX		
	6 MV‐FF	***109.5 ± 11.8***	71.5 ± 12.1
	6 MV‐FFF	***115.9 ± 12.1***	62.7 ± 14.1
	10 MV‐FF	***103.5 ± 8.6***	68.1 ± 7.2
	10 MV‐FFF	***112.1 ± 15.3***	69.0 ± 10.7

RPM, real‐time positioning management; FF, flattened; FFF = flattening‐filter‐free.

The incorrected data have been removed from the results. Additionally, correct data have been added to generate a new Table 1 (shown below). The specific values for Δt_gate‐on_ and Δt_beam‐on_ have got smaller, but all of the original findings and conclusions remain valid.

The new corrected table 1 values have resulted in the following changes (***bold italics***) to the text of the manuscript.

**Abstract**

The total time delay between TrueBeam and SyncTraX was <***200 ms*** for each photon beam.

**Results**

**B. Evaluation of time delay using oscilloscope**

The mean ± SD values of Δt_gate‐on_ were ***64.2 ± 11.6 ms, 70.3 ± 12.2 ms, 58.3 ± 8.6 ms,*** and ***66.4 ± 15.4 ms*** in the case of 6 MV‐FF, 6 MV‐FFF, 10 MV‐FF, and 10 MV‐FFF photon beams, respectively.

For each photon beam, Δt_beam‐on_ + Δt_beam‐off_ <***200 ms***.

**Discussion**

In this study, the time delay was measured using an oscilloscope, and the total time delay was <***200*** **ms** for each photon beam.
