# Supplementary material for: Evaluation of a combined respiratory‐gating system comprising the TrueBeam linear accelerator and a new real‐time tumor‐tracking radiotherapy system: a preliminary study
Source: J Appl Clin Med Phys. 2016 Jul 8;17(4):202–13. doi: 10.1120/jacmp.v17i4.6114 (PMC5690064; doi:10.1120/jacmp.v17i4.6114)
Supplement: Supplementary file 1 — Supplementary Material [file ACM2-17-202-s001.pdf]

CERTIFICATE OF ENGLISH EDITING

This document certifies that the paper listed below has been edited to ensure that the language is clear and free of errors. The logical presentation of ideas and the structure of the paper were also checked during the editing process. The edit was performed by professional editors at Editage, a division of Cactus Communications. The intent of the author's message was not altered in any way during the editing process. The quality of the edit has been guaranteed, with the assumption that our suggested changes have been accepted and have not been further altered without the knowledge of our editors.

Title Of The Paper

Evaluation of a combined respiratory gating system comprising the TrueBeam linear accelerator and a new real-time tumor-tracking radiotherapy system

Authors

Takehiro Shiinoki

Job Code

MDPKX_1_3

Signature

Nikesh Gosalia,
Vice President, Author Services, Editage

Date of Issue
January 15, 2016

Editage, a brand of Cactus Communications, offers professional English language editing and publication support services to authors engaged in over 500 areas of research. Through its community of experienced editors, which includes doctors, engineers, published scientists, and researchers with peer review experience, Editage has successfully helped authors get published in internationally reputed journals. Authors who work with Editage are guaranteed excellent language quality and timely delivery.

Contact Editage

Worldwide	Japan	Korea	China	Brazil	Taiwan
request@editage.com	submissions@editage.com	submit-korea@editage.com	fabiao@editage.cn	inquiry.brazil@editage.com	submitjobs@editage.com
+1 877-334-8243	+81 03-6868-3348	1544-9241	400-005-6055	0800-892-20-97	02 2657 0306
www.editage.com	www.editage.jp	www.editage.co.kr	www.editage.cn	www.editage.com.br	www.editage.com.tw
